# Impact of COVID-19 restrictions on thyroid cancer diagnoses in a comprehensive cancer center (2017–2023)

**DOI:** 10.1371/journal.pone.0340347

**Published:** 2026-01-15

**Authors:** Hayley Mann, Erin Dimon, Yichi Zhang, Natalia Arroyo, Abdullah Adil, Yanchen Zhang, Oguzhan Alagoz, Sara Fernandes-Taylor, Erin J. Aiello Bowles, Louise Davies, David O. Francis

**Affiliations:** 1 Department of Otolaryngology Head & Neck Surgery, University of Wisconsin School of Medicine and Public Health, Madison Wisconsin, United States of America; 2 Department of Industrial and Systems Engineering, College of Engineering, University of Wisconsin-Madison, Madison, Wisconsin, United States of America; 3 UW Carbone Cancer Center, School of Medicine and Public Health, University of Wisconsin-Madison, Madison, Wisconsin, United States of America; 4 Department of Otolaryngology-Head and Neck Surgery, University of Michigan, Ann Arbor, Michigan, United States of America; 5 Department of Medicine, University of Wisconsin School of Medicine and Public Health, Madison Wisconsin, United States of America; 6 Kaiser Permanente Washington Health Research Institute, Kaiser Permanente Washington, Seattle, Washington, United States of America; 7 The VA Outcomes Group, Department of Veterans Affairs Medical Center, White River Junction, Vermont, United States of America; 8 Section of Otolaryngology, Geisel School of Medicine at Dartmouth, Hanover, New Hampshire, United States of America; 9 The Dartmouth Institute for Health Policy and Clinical Practice, Lebanon, New Hampshire, United States of America; Hamad Medical Corporation, QATAR

## Abstract

**Introduction:**

The Coronavirus 2019 (COVID-19) pandemic disrupted healthcare delivery across the United States, leading to a sharp decline in outpatient visits. Given that thyroid nodule evaluation and diagnosis typically occur in outpatient settings, we hypothesized that these disruptions would reduce thyroid cancer incidence. This study evaluates trends in thyroid cancer diagnosis, treatment, and outcomes at a U.S. comprehensive cancer center from 2017 to 2023.

**Methods:**

We included adults with pathologically confirmed thyroid cancer diagnosis between January 1, 2017, and December 31, 2023. Primary analyses assessed (1) annual percent change (APC) in thyroid cancer incidence from 2017–2023 and (2) incidence trends across three periods: pre-pandemic (Jan 2017–Mar 2020), pandemic (Apr 2020–Mar 2021), and COVID-years (Apr 2021–Dec 2023). Annual incidence rates were calculated using the total number of individuals cared for within our health system each year as the denominator. Secondary outcomes included tumor size, extent and timing of surgery, and 1-year all-cause mortality across time periods.

**Results:**

Among 1,031 patients (68% women, 92% White, mean age 52 ± 15.5), age- and sex-adjusted thyroid cancer incidence declined from 34 to 15.6 per 100,000 persons between 2017 and 2022, with an average APC of –14.4%. Diagnoses rose slightly in 2023 (21 per 100,000) compared to 2022 but remained 38% below 2017 levels. No significant differences were observed in tumor size, surgical treatment, gender ratio, or 1-year all-cause mortality across timeframes.

**Conclusion:**

Age- and sex-adjusted annual incidence of thyroid cancer declined steadily from 2017 to 2022. The annual decrease in thyroid cancer continued through the pandemic years. Tumor size and mortality remained stable across all years evaluated. The increase in diagnoses noted in 2023 may be a correction due to missed cases during the years of the pandemic or could represent a new steady state incidence after years of decline.

## Introduction

The Coronavirus 2019 (COVID) pandemic imposed burdens on healthcare systems worldwide. Shortly after the declaration of a national emergency in the United States (US) on March 13, 2020, most US healthcare centers restricted services to maximize hospital and intensive care unit (ICU) capacities for incoming COVID patients and prioritized care of high acuity medical conditions [1–3]. Specific actions included reducing the number of clinics, redeployment of staff to COVID-related work, and a shift to a telemedicine visit model. Chronic and preventative care was postponed, as were non-urgent diagnostic tests and low-to-intermediate acuity procedures. Consequently, outpatient care visits declined by as much as 60% between March 2020 and September 2020 relative to previous years [4–7].

With the decline in outpatient services utilization, outpatient imaging declined by as much as 68% [8]. Thyroid nodules and cancers are largely detected incidentally through cross-sectional imaging of the neck and chest regions done for other reasons or via a referral for thyroid ultrasound by a primary care provider [9,10]. We hypothesized that the reduction in outpatient visits and cross-sectional imaging during COVID restrictions would reduce thyroid cancer incidence rates at the time of the initial pandemic onset and shut-down, followed by a rebound increase in new diagnoses after many restrictions were lifted and services re-opened in the fall of 2020.

The literature describes potential downstream consequences of delays in thyroid cancer diagnosis and treatment [11–13]. To date, US and international studies have described the effect of COVID-era restrictions on the rate of thyroid cancer surgery [14–16]. However, the longer-term impact of COVID-related disruptions in healthcare delivery on thyroid cancer diagnoses prior to, during, and following initial pandemic restrictions has not been well described [17,18]. The present study evaluates how thyroid cancer diagnosis, treatment, and outcomes in a large, tertiary US health system were altered in the three years after the onset of the pandemic.

## Methods

### Setting

This observational study, approved by the University of Wisconsin (UW) Health Science Institutional Review Board (#2021−0601), used data from the electronic health record and the University of Wisconsin Carbone Cancer Center (UWCCC), a National Cancer Institute-designated comprehensive cancer center. Data was extracted on February 28, 2025; authors did not have access to information that could identify participants during or after data collection. The UWCCC is part of UW Health, the integrated health system of the University of Wisconsin-Madison that includes 7 hospitals and more than 80 clinics across Wisconsin. Over 30,000 people seek care for cancer diagnoses, treatment, and long-term follow-up at the UWCCC annually.

### Inclusion criteria

Clinical data were extracted from the UWCCC cancer registry, including variables defined by the National Cancer Institute’s Surveillance, Epidemiology, and End Results (SEER) program and regularly reported to the Wisconsin state cancer registry. Adults (aged ≥18 years) who were diagnosed with a first pathologically confirmed thyroid cancer (ICD-O-3 code C73.9) between January 1, 2017 and December 31, 2023 were included. Patient data from 2017–2019 was included to evaluate the state of thyroid cancer diagnoses in the pre-pandemic period. The sample of patients was linked to sociodemographic information and service area in the electronic health record using unique patient identifiers and date of diagnosis. Patients who did not receive routine care within the UW Health service area (as defined by place of residence in proximity to the service area) were excluded.

### Data extracted

Data for the final cohort were extracted from the electronic health record and cancer registry. These data included sex, age, race (White, Asian, Native American or Alaska Native, Black or African American, Native Hawaiian or other Pacific Islander), ethnicity (Hispanic/Latino), cancer diagnosis date, surgery date, extent of surgery (e.g., total thyroidectomy, lobectomy), pathologic tumor size (cm), and 1-year, all-cause mortality.

The total number of individuals cared for at UW Health each year was extracted from the medical records and used as the denominator to calculate annual incidence rates from 2017–2023. To be included in the total, the individual must have had at least one completed encounter within UW Health and reside within the UW Health primary service area in the designated calendar year. Because the UWCCC is the only comprehensive cancer center in Wisconsin and a regional referral center with limited specialty surgery competition, it is uncommon for patients diagnosed with thyroid cancer at the UWCCC to have their surgery elsewhere.

### Statistical analysis

The primary outcome was thyroid cancer incidence (i.e., new thyroid cancer diagnoses per 100,000 persons). The overall and sex-stratified annual incidence rates were calculated by dividing the number of new cancers by the total number of UW Health individuals per year and multiplying the raw rate by 100,000. Incidence rates were compared in two separate analyses. First, we evaluated the stability of overall thyroid cancer incidence rates prior to COVID restrictions. Baseline annual percent changes (APCs) in incidence rates were calculated as the slope of the line of two consecutive annual rates on a log scale [19,20]. We used analysis of covariance to determine differences in the annual change in incidence rates from 2017 to 2023 adjusted for sex and age at diagnosis. Those missing data on sex were excluded from the sex- and age-adjusted analysis. Second, we evaluated changes that occurred pre-pandemic, pandemic, and COVID-years thereafter (January 2017-March 2020; April 2020-March 2021; April 2021-December 2023 respectively). Two-sample t-tests compared incidence rates during these time frames as well as annual incidence rates. Monthly incidence rates were also calculated by determining the number of new diagnoses occurring each month. To account for varying population sizes across years, monthly incidence rates were also standardized per 100,000 individuals using the total number of distinct patients diagnosed in each corresponding year.

Secondary outcomes of this study included tumor size, treatment patterns (e.g., extent and time to surgery), and all-cause mortality. Patients were followed via the electronic medical record for 1 year after their diagnosis date to evaluate the date and extent of surgery (lobectomy CPT codes: 60210, 60212, 60220, 60225; total thyroidectomy CPT codes: 60240, 60252, 60254, 60270, 60271) and vital status. Because there currently are no guidelines for appropriate time to surgery, we set the 1-year follow-up period to reflect current trends: 98% of thyroid cancer patients are treated within 180 days of diagnosis and receive follow-up care 6–12 months after diagnosis [12,21]. Due to the inherent nature of retrospective chart reviews, some secondary outcomes data were missing but those patients were still included in the overall incidence analysis as long as they met the inclusion criteria. Two-sample t-tests were used to compare mean pathologic size, time to surgery, extent of surgery, and 1-year all-cause mortality rates between pre-pandemic, pandemic, and COVID-years periods. Pearson’s chi-squared tests of independence were used to determine if there was an association between these timeframes and (1) extent of surgery and (2) all-cause mortality rates. Statistical analyses were done using SAS version 9.4.

## Results

### Study population

In all, 1,031 patients met inclusion criteria (68% women, mean age 51.6 years ± 15.5, 92% White, 95% not Hispanic or Latino; Table 1). Patient demographics were similar across all years (2017–2023). 90% of patients diagnosed with thyroid cancer had surgery within 1 year of diagnosis (N = 932). Total thyroidectomy was performed in 61% of patients (n = 572), lobectomy in 36% (n = 339), and other surgeries (e.g., completion thyroidectomy) in 2% (n = 21). The 1-year, all-cause mortality was 4% (n = 36). Women composed 68% of pre-pandemic thyroid cancer diagnoses (359 of 532), 72% of pandemic diagnoses (101 of 140), and 67% of COVID-years diagnoses (242 of 359) (Table 1).

**Table 1:** Study population characteristics (2017-2023)*.

	All (n = 1,031)	Pre-pandemic (n = 532)	Pandemic (n = 140)	COVID-years (n = 359)
**Age: Mean (SD)**	51.6 (15.5)	51.6 (15.3)	50.9 (15.5)	51.7 (15.9)
**Sex**		**n (%)**	**n (%)**	**n (%)**
Female	702 (68.1)	359 (67.5)	101 (72.1)	242 (67.4)
Male	329 (31.9)	173 (32.5)	39 (27.9)	117 (32.6)
**Race**				
White	951 (92.3)	494 (92.9)	131 (93.6)	326 (91.3)
Black or African American	31 (3.0)	18 (3.4)	3 (2.1)	10 (2.8)
Asian	25 (2.4)	9 (1.7)	4 (2.9)	12 (3.4)
Native Hawaiian or Other Pacific Islander	2 (0.2)	2 (0.4)	0 (0)	0 (0)
American Indian or Alaskan Native	5 (0.5)	2 (0.4)	0 (0)	3 (0.8)
Patient declined/unavailable	17 (1.6)	7 (1.3)	4 (2.8)	6 (1.7)
**Ethnicity**				
Not Hispanic	982 (95.3)	505 (94.9)	136 (97.1)	341 (95.0)
Hispanic	39 (3.8)	21 (4.0)	4 (2.9)	14 (3.9)
Patient declined/unavailable	10 (1.0)	7 (1.1)	0 (0)	4 (1.1)
**Subtype**				
Anaplastic	14 (1.4)	5 (0.9)	3 (2.1)	6 (1.7)
Follicular	29 (2.8)	16 (3.0)	7 (5.0)	6 (1.7)
Hurthle	16 (1.6)	6 (1.1)	2 (1.4)	8 (2.2)
Medullary	11 (1.1)	4 (0.8)	2 (1.4)	5 (1.4)
Papillary	342 (33.2)	189 (35.5)	35 (25.0)	118 (32.9)
Unclassified	619 (60.0)	312 (58.7)	91 (65.0)	216 (60.2)
**Extent of surgery within one year of diagnosis**	n = 932	n = 471	n = 127	n = 334
Total thyroidectomy	572 (61.4)	304 (64.5)	75 (59.1)	193 (57.8)
Lobectomy	339 (36.4)	154 (32.7)	50 (39.4)	135 (40.4)
Other (e.g., completion thyroidectomy)	21 (2.3)	13 (2.8)	2 (1.6)	6 (1.8)
**All-cause mortality within one year of diagnosis**				
Alive	896 (96.1)	447 (94.9)	119 (93.7)	330 (98.8)
Dead	36 (3.9)	24 (5.1)	8 (6.3)	4 (1.2)

*Pre-pandemic 1/1/2017–3/31/2020; Pandemic 4/1/2020–3/31/2021; COVID-years 4/1/2021-12/31/2023.

### Annual incidence rates across timeframes

**Fig 1:**
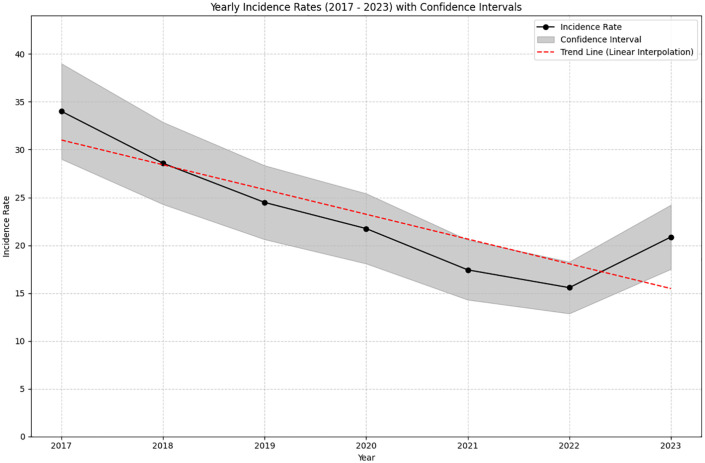
Thyroid cancer incidence rates from 2017-2023 UW Health patients.

Age- and sex-adjusted annual incidence of thyroid cancer declined steadily from 2017 to 2022 (−14.4 average annual percent change [APC]; Table 2, Fig 1). Mean pre-pandemic (2017–2019), pandemic (2020–2021), and one year thereafter (2022) annual incidence declined at similar rates. After 2022, the incidence rate of new thyroid cancer diagnoses increased (21 in 2023 vs 16 in 2022 per 100,000 persons), but remained 38% lower than that observed in 2017 (21 vs 34 per 100,000 persons).

**Table 2:** Average annual percent change in baseline thyroid cancer incidence rates from 2017-2023.

Year	Incidence rate per 100,000 persons (95% CI)	APC (%)
2017	34.0 (29.0–39.0)	n/a
2018	28.6 (24.3 - 32.9)	−16.0
2019	24.5 (20.6 - 28.3)	−14.3
2020	21.7 (18.1–25.4)	−11.2
2021	17.4 (14.3- 20.6)	−19.6
2022	15.6 (12.9- 18.3)	−10.7
2023	20.9 (17.5–24.2)	34.0

### Monthly incidence rates across timeframes

The average monthly incidence rate of thyroid cancer declined 37% from pre-pandemic to COVID years (pre-pandemic: 2.36 per 100,000, pandemic: 1.84 per 100,000, COVID years: 1.48 per 100,000; respectively) (Table 3). Average monthly incidence among women and men each declined 37% between pre-pandemic and COVID years (women: pre-pandemic: 2.96 per 100,000, pandemic: 2.45 per 100,000, and COVID-years: 1.84 per 100,000; respectively; men: pre-pandemic: 1.66 per 100,000, pandemic: 1.11 per 100,000, and COVID years 1.05 per 100,000).

**Table 3:** Comparison of monthly incidence rates between pre-pandemic, pandemic, and COVID-years timeframes.

	Pre-pandemic	Pandemic	COVID-years
Overall – average monthly incidence rate per 100,000 (95% CI)	2.36	1.84	1.48
Men – average monthly incidence rate per 100,000 (95% CI)	1.66	1.11	1.05
Women – average monthly incidence rate per 100,000 (95% CI	2.96	2.45	1.84

### Effect of COVID maximum restriction

During maximum COVID restrictions from March through June 2020, there was a 41% decrease in average incidence rates compared to the same months in 2019 (Table 4). However, this short-term decline was followed by an increase in monthly incidence rates later in the year, resulting in no overall change in the trend of annual thyroid cancer incidence in 2020.

**Table 4:** Monthly thyroid cancer incidence rates during 2020.

Month of Diagnosis	Monthly Incidence rate per 100,000 persons (95% CI)
1	2.09 (0.96-3.23)
2	1.77 (0.72-2.82)
3*	0.97 (0.19-1.74)
4*	0.48 (−0.06-1.03)
5*	1.29 (0.40-2.18)
6*	1.77 (0.72-2.82)
7	1.45 (0.50-2.40)
8	2.26 (1.07-3.44)
9	1.93 (0.84-3.03)
10	2.42 (1.19-3.64)
11	2.42 (1.19-3.64)
12	2.90 (1.56-4.24)

*= Maximum COVID restrictions (March-June 2020).

### Tumor size, surgery extent and timing, and all-cause mortality

From 2017–2023, mean tumor size was 2.1 cm (SD 1.6) and did not vary across timeframes (Table 5). Pre-pandemic mean time to surgery after diagnosis was 24.8 days (SD 42.5). The average time to surgery did not vary during the pandemic timeframe compared to pre-pandemic times (Table 5). However, we did observe a significantly increased wait time for surgery during COVID-years (35.8 days ± 50.9) as compared to pre-pandemic (24.8 days ± 42.5), p < 0.001) and pandemic timeframes (24.1 days ± 51.5), p < 0.03) (Table 5). Extent of surgery and 1-year all-cause mortality rates did not differ across periods (Table 1).

**Table 5:** Comparison of tumor characteristics, extent of surgery, and survival during baseline and COVID periods (secondary outcomes).

	All	Pre-pandemic (n = 471)	Pandemic (n = 127)	COVID-years (n = 334)
**Tumor size**				
Mean tumor size (SD), cm	2.1 (1.6)	2.1 (1.6)	2.2 (1.7)	2.1 (1.6)
Median tumor size (IQR), cm	1.7 (0.9-3.0)	1.6 (0.9-3.0)	1.6 (0.8-3.4)	1.8 (1.0-3.0)
**Time to surgery**				
Mean time to surgery (SD), days	28.6 (47.2)	24.8 (42.5)	24.1 (51.5)*	35.8 (50.9)**

*p ≤ .001 comparing Pre-pandemic vs COVID years.

**p ≤ .05 comparing Pandemic vs COVID years.

## Discussion

Thyroid nodules are predominantly detected in an outpatient setting either incidentally on imaging or are referred to radiology by primary care providers for concerning symptoms or physical exam findings [9,10,22]. COVID restrictions severely reduced in-person primary care visits, decreased diagnostic testing, and decreased performance of low-to-moderate acuity procedures and surgeries [5,6]. We, therefore, hypothesized that COVID restrictions would result in a reduced incidence of thyroid cancer. While we observed a short-term reduction in incidence during the three-months of maximum COVID restrictions, the overall downward trend in annual thyroid cancer incidence rates continued between 2017 and 2022 (average APC −14.4%) annually in our health system. There was an observable increase in incidence in 2023. Notably, across all time periods, there was no observable change in mean thyroid tumor size or 1-year all-cause mortality. Finally, we observed a shorter time to surgery during the pandemic than during the COVID-years timeframe. This difference can be explained by operating room availability. During the pandemic all elective surgical cases were restricted while cancer surgeries were prioritized. Therefore, there was more operating room availability for cancer cases because of the elective surgery restrictions.

### Thyroid cancer incidence and COVID

Consistent with prior studies, we found thyroid cancer incidence did decline substantially during the height of COVID restrictions (March-May 2020) [17,18,23]. In fact, average rates during this time dropped 41% compared to similar months in 2019. However, this short-term decline had no impact on the overall 2020 annual incidence of thyroid cancer. Thyroid cancer incidence steadily declined by 14.4% annually from 2017 to 2022, followed by an increase in 2023. The observed increase in 2023 following several years of a steady decline may reflect a delayed rebound effect resulting from the prolonged disruption caused by the COVID pandemic and the likely required additional time it took patients and healthcare systems to fully normalize. Thus, this increase could be attributed to a multitude of confluent factors such as increased appointment availability, heightened medical awareness, greater healthcare-seeking behavior, and resumed or even increased routine care. Of note, even with a slight increase in incidence in 2023, the rate was still 38% lower than observed in 2017.

Prior studies have consistently shown a plateauing of thyroid cancer incidence from 2015 to 2019 [24–26]. More recently, research has shown that thyroid cancer incidence decreased during early COVID years from 2020–2021 [23]; however, the present study is the first to show data from 2022–2023. Reasons for declining thyroid cancer incidence between 2017 and 2022 are likely multifactorial and not fully elucidated. One explanation could be improved guidance from the American Thyroid Association (ATA) and American College of Radiology (ACR) about when to biopsy nodules [27,28]. The American Thyroid Association Guidelines Task Force Guideline-adherent biopsy recommendations were introduced in 2015 to improve FNA accuracy and decrease unnecessary biopsies at a time when thyroid cancer diagnoses had tripled over the previous two decades, almost entirely secondary to clinically occult cancers [27,29]. In 2017 ACR TIRADS was introduced, an ultrasound risk stratification system to provide systematic guidance to radiologists to consider when to biopsy a nodule [29]. It has been hypothesized that the confluence of guidance from these prominent national organizations promoted improved decision-making about when to biopsy thyroid nodules and therefore reduced the number of new thyroid cancer diagnoses. Another theory is that the reservoir of thyroid cancers has gotten smaller. Based on autopsy studies, it estimated that 10% of the population has occult thyroid cancer [30]. If this reservoir of undetected cancers was depleted due to more thyroid surgeries or other reasons, then fewer cancers will be discovered and treated. Further studies are needed to understand the reasons for the steady decline; however, it is clear that at our center, the COVID pandemic did not significantly alter the trajectory.

### Tumor size and extent of surgery

Detection of small, non-palpable nodules leads to 23–51% of papillary thyroid cancer diagnoses, which are 4.3 times more common in women than men [31,32]. We anticipated that less and delayed detection during the COVID pandemic would lead to more and larger tumors being diagnosed when in-person outpatient visits and imaging referrals normalized. This was not observed. Instead, our data demonstrated a consistent mean tumor size of 2.1 cm across all time periods considered (pre-pandemic, pandemic, and COVID-years; **Table 5**).

### Limitations

This was a single institution study from a tertiary care center in the Midwest; therefore, findings may not be generalizable to other institutions. Similarly, the practice patterns among our primary care physicians, radiologists, endocrinologists, and endocrine surgeons and our institution’s response to COVID may differ from other centers, which would also affect generalizability. Future multi-institutional or national studies of the long-term effects of COVID restrictions on thyroid cancer diagnoses would provide further insight as to the trends in the rate of thyroid cancer diagnosis outside of an academic medical center. Unique to this study is the continued, steady decline in thyroid cancer incidence over a five-year period followed by a single year of increased incidence rates. While we offer possible hypotheses for these findings, it deserves further investigation. It will also be important to track thyroid cancer incidence beyond 2023 to determine if the observed downward trend will continue and what consequence the decline in rates of thyroid cancer will have on patient morbidity, mortality, and overall healthcare costs.

## Conclusions

We report a consistent declining trend in thyroid cancer incidence from 2017–2022 at our tertiary care center, with evidence of increasing incidence in 2023. Limitations imposed by the COVID pandemic did not significantly alter the trajectory of this decline. Thyroid cancer size at diagnosis, extent of surgery, and 1-year all-cause mortality were unaffected by the reduced detection of thyroid cancer in our health system. Time will determine the durability of this decline, whether there will be normalization of rates to pre-pandemic levels, and the effect this will have on patient population and health systems.

## References

[1] Centers for Medicare & Medicaid Services. Non-Emergent, Elective Medical Services, and Treatment Recommendations. www.cms.gov/files/document/cms-non-emergentelective-medical-recommendations.pdf. Accessed 2025 August 6.

[2] Dichter JR, Devereaux AV, Sprung CL, Mukherjee V, Persoff J, Baum KD, et al. Mass critical care surge response during COVID-19: Implementation of contingency strategies - a preliminary report of findings from the task force for mass critical care. Chest. 2022;161(2):429–47. doi: 10.1016/j.chest.2021.08.072 34499878 PMC8420082

[3] Hick JL, Einav S, Hanfling D, Kissoon N, Dichter JR, Devereaux AV, et al. Surge capacity principles: care of the critically ill and injured during pandemics and disasters: CHEST consensus statement. Chest. 2014;146(4 Suppl):e1S-e16S. doi: 10.1378/chest.14-0733 25144334

[4] Chatterji P, Li Y. Effects of the COVID-19 pandemic on outpatient providers in the United States. Med Care. 2021;59(1):58–61. doi: 10.1097/MLR.0000000000001448 33136711

[5] Mehrotra ACM, Linetsky D, Hatch H, Cutler DM, Schneider EC. The Impact of COVID-19 on Outpatient Visits in 2020: Visits Remained Stable, Despite a Late Surge in Cases. Commonwealth Fund. 2021. https://www.commonwealthfund.org/publications/2021/feb/impact-covid-19-outpatient-visits-2020-visits-stable-despite-late-surge

[6] Patel SY, Mehrotra A, Huskamp HA, Uscher-Pines L, Ganguli I, Barnett ML. Trends in outpatient care delivery and telemedicine during the COVID-19 pandemic in the US. JAMA Intern Med. 2021;181(3):388–91. doi: 10.1001/jamainternmed.2020.5928 33196765 PMC7670397

[7] Qian L, Sy LS, Hong V, Glenn SC, Ryan DS, Morrissette K, et al. Disparities in outpatient and telehealth visits during the COVID-19 pandemic in a large integrated health care organization: retrospective cohort study. J Med Internet Res. 2021;23(9):e29959. doi: 10.2196/29959 34351865 PMC8412134

[8] Parikh KD, Ramaiya NH, Kikano EG, Tirumani SH, Pandya H, Stovicek B, et al. COVID-19 pandemic impact on decreased imaging utilization: a single institutional experience. Acad Radiol. 2020;27(9):1204–13. doi: 10.1016/j.acra.2020.06.024 32665091 PMC7340053

[9] Davenport C, Alderson J, Yu IG, Magner AC, M O’Brien D, Ghiollagain MN, et al. A review of the propriety of thyroid ultrasound referrals and their follow-up burden. Endocrine. 2019;65(3):595–600. doi: 10.1007/s12020-019-01920-1 30955175

[10] Davies L, Morris LGT, Haymart M, Chen AY, Goldenberg D, Morris J, et al. American association of clinical endocrinologists and american college of endocrinology disease state clinical review: the increasing incidence of thyroid cancer. Endocr Pract. 2015;21(6):686–96. doi: 10.4158/EP14466.DSCR 26135963 PMC4923940

[11] Epic Research. Delayed cancer screenings. https://epicresearch.org/articles/delays-in-preventive-cancer-screenings-during-covid-19-pandemic/. 2020. Accessed 2025 August 11.

[12] Fligor SC, Lopez B, Uppal N, Lubitz CC, James BC. Time to surgery and thyroid cancer survival in the United States. Ann Surg Oncol. 2021;28(7):3556–65. doi: 10.1245/s10434-021-09797-z 33768394

[13] Mast C, MdR A. Delayed cancer screenings - a second look. https://epicresearch.org/articles/delayed-cancer-screenings-a-second-look/. 2020. Accessed 2025 August 11.

[14] Beninato T, Laird AM, Graves CE, Drake FT, Alhefdhi A, Lee JA, et al. Impact of the COVID-19 pandemic on the practice of endocrine surgery. Am J Surg. 2022;223(4):670–5. doi: 10.1016/j.amjsurg.2021.07.009 34315576 PMC8294714

[15] Kim SH, Min E, Hwang YM, Choi YS, Yi JW. Impact of COVID-19 Pandemic on Thyroid Surgery in a University Hospital in South Korea. Cancers (Basel). 2022;14(17):4338. doi: 10.3390/cancers14174338 36077872 PMC9454546

[16] Medas F, Ansaldo GL, Avenia N, Basili G, Bononi M, Bove A, et al. Impact of the COVID-19 pandemic on surgery for thyroid cancer in Italy: nationwide retrospective study. Br J Surg. 2021;108(4):e166–7. doi: 10.1093/bjs/znab012 33659983 PMC7989577

[17] Nickel B, Glover A, Miller JA. Delays to low-risk thyroid cancer treatment during COVID-19-refocusing from what has been lost to what may be learned and gained. JAMA Otolaryngol Head Neck Surg. 2021;147(1):5–6. doi: 10.1001/jamaoto.2020.3878 33119084

[18] Nickel B, Miller JA, Cvejic E, Gild ML, Cope D, Dodd R, et al. Thyroid cancer clinicians’ views and experiences of delayed treatment during the COVID-19 pandemic: an international cross-sectional survey. ANZ J Surg. 2021;91(12):2562–4. doi: 10.1111/ans.17128 34350698 PMC8420195

[19] Fay MP, Tiwari RC, Feuer EJ, Zou Z. Estimating average annual percent change for disease rates without assuming constant change. Biometrics. 2006;62(3):847–54. doi: 10.1111/j.1541-0420.2006.00528.x 16984328

[20] Yan KL, Li S, Tseng C-H, Kim J, Nguyen DT, Dawood NB, et al. Rising Incidence and incidence-based mortality of thyroid cancer in California, 2000-2017. J Clin Endocrinol Metab. 2020;105(6):dgaa121. doi: 10.1210/clinem/dgaa121 32166320

[21] Patel KN, Yip L, Lubitz CC, Grubbs EG, Miller BS, Shen W, et al. The American association of endocrine surgeons guidelines for the definitive surgical management of thyroid disease in adults. Ann Surg. 2020;271(3):e21–93. doi: 10.1097/SLA.0000000000003580 32079830

[22] Lincango-Naranjo E, Solis-Pazmino P, El Kawkgi O, Salazar-Vega J, Garcia C, Ledesma T, et al. Triggers of thyroid cancer diagnosis: a systematic review and meta-analysis. Endocrine. 2021;72(3):644–59. doi: 10.1007/s12020-020-02588-8 33512656

[23] Bell R, Weinberger DM, Venkatesh M, Fernandes-Taylor S, Francis DO, Davies L. Thyroid cancer incidence during 2020 to 2021 COVID-19 variant waves. JAMA Otolaryngol Head Neck Surg. 2024. 10.1001/jamaoto.2024.3146 39388144 PMC11581733

[24] ChenMM, LuuM, SacksWL, OrloffL, WallnerLP, ClairJM-S, et al. Trends in incidence, metastasis, and mortality from thyroid cancer in the USA from 1975 to 2019: a population-based study of age, period, and cohort effects. Lancet Diabetes Endocrinol. 2025;13(3):188–95. doi: 10.1016/S2213-8587(24)00310-3 39922210

[25] Li Y, Che W, Yu Z, Zheng S, Xie S, Chen C, et al. The incidence trend of papillary thyroid carcinoma in the united states during 2003-2017. Cancer Control. 2022;29:10732748221135447. doi: 10.1177/10732748221135447 36256588 PMC9583193

[26] Powers AE, Marcadis AR, Lee M, Morris LGT, Marti JL. Changes in trends in thyroid cancer incidence in the United States, 1992 to 2016. JAMA. 2019;322(24):2440–1. doi: 10.1001/jama.2019.18528 31860035 PMC6990659

[27] Haugen BR, Alexander EK, Bible KC, Doherty GM, Mandel SJ, Nikiforov YE, et al. 2015 American thyroid association management guidelines for adult patients with thyroid nodules and differentiated thyroid cancer: the American thyroid association guidelines task force on thyroid nodules and differentiated thyroid cancer. Thyroid. 2016;26(1):1–133. doi: 10.1089/thy.2015.0020 26462967 PMC4739132

[28] Tessler FN, Middleton WD, Grant EG, Hoang JK, Berland LL, Teefey SA, et al. ACR thyroid imaging, reporting and data system (TI-RADS): white paper of the ACR TI-RADS committee. J Am Coll Radiol. 2017;14(5):587–95. doi: 10.1016/j.jacr.2017.01.046 28372962

[29] Aschebrook-Kilfoy B, Schechter RB, Shih Y-CT, Kaplan EL, Chiu BC-H, Angelos P, et al. The clinical and economic burden of a sustained increase in thyroid cancer incidence. Cancer Epidemiol Biomarkers Prev. 2013;22(7):1252–9. doi: 10.1158/1055-9965.EPI-13-0242 23677575

[30] Arroyo N, Bell KJL, Hsiao V, Fernandes-Taylor S, Alagoz O, Zhang Y, et al. Prevalence of subclinical papillary thyroid cancer by age: meta-analysis of autopsy studies. J Clin Endocrinol Metab. 2022;107(10):2945–52. doi: 10.1210/clinem/dgac468 35947867 PMC9516102

[31] Haymart MR, Banerjee M, Reyes-Gastelum D, Caoili E, Norton EC. Thyroid Ultrasound and the Increase in diagnosis of low-risk thyroid cancer. J Clin Endocrinol Metab. 2019;104(3):785–92. doi: 10.1210/jc.2018-01933 30329071 PMC6456891

[32] LeClair K, Bell KJL, Furuya-Kanamori L, Doi SA, Francis DO, Davies L. Evaluation of gender inequity in thyroid cancer diagnosis: differences by sex in US thyroid cancer incidence compared with a meta-analysis of subclinical thyroid cancer rates at autopsy. JAMA Intern Med. 2021;181(10):1351–8. doi: 10.1001/jamainternmed.2021.4804 34459841 PMC8406211

